# Impacts of COVID-19 on Air Quality through Traffic Reduction

**DOI:** 10.3390/ijerph19031718

**Published:** 2022-02-02

**Authors:** Hyemin Hwang, Jae Young Lee

**Affiliations:** Environmental and Safety Engineering Department, Ajou University, Suwon 16499, Korea; hhm8866@ajou.ac.kr

**Keywords:** COVID-19, social distancing, traffic, air quality, Seoul, generalized additive model

## Abstract

In 2020, the first case of COVID-19 was confirmed in Korea, and social distancing was implemented to prevent its spread. This reduced the movement of people, and changes in air quality were expected owing to reduced emissions. In the present paper, the impact of traffic volume change caused by COVID-19 on air quality in Seoul, Korea, is examined. Two regression analyses were performed using the generalized additive model (GAM), assuming a Gaussian distribution; the relationships between (1) the number of confirmed COVID-19 cases in 2020–2021 and the rate of change in the traffic volume in Seoul, and (2) the traffic volume and the rate of change in the air quality in Seoul from 2016 to 2019 were analyzed. The regression results show that traffic decreased by 0.00431% per COVID-19 case; when traffic fell by 1%, the PM_10_, PM_2.5_, CO, NO_2_, O_3_, and SO_2_ concentrations fell by 0.48%, 0.94%, 0.39%, 0.74%, 0.16%, and −0.01%, respectively. This mechanism accounts for air quality improvements in PM_10_, PM_2.5_, CO, NO_2_, and O_3_ in Seoul during 2020–2021. From these results, the majority of the reduction in pollutant concentrations in 2020–2021 appears to be the result of a long-term declining trend rather than COVID-19.

## 1. Introduction

In December 2019, a novel coronavirus (COVID-19) was first reported in Wuhan, China [1]. COVID-19 has spread to more than 210 countries and territories [2], and on 11 March 2020, the World Health Organization declared COVID-19 a pandemic [3]. As of 29 June 2021, the cumulative number of COVID-19 cases worldwide reached more than 180 million, and the cumulative number of deaths was nearly 4 million [4].

Many countries have implemented measures, such as lockdowns, to prevent the spread of COVID-19, including regulations on social distancing that limit human activities [5]. These measures have had an impact on improving air quality by reducing the emissions of pollutants from human activities [6]. The decreases in surface NO_2_ and PM_2.5_ concentrations, and increases in O_3_ concentrations in 34 countries were reported [7]. Several studies evaluated the effect of lockdown on the air quality in each country. In the United States, during the lockdown period, NO_2_ and CO decreased by 49% and 37%, respectively, from 2017 to 2019. This trend increased with the increase in the local population density [8]. In Seattle, Washington, roadside (near I-5 Express) pollutant concentrations of BC, PM_2.5_, NO, NO_2_, NO_x_, and CO decreased by 25%, 33%, 33%, 29%, 30%, and 17%, respectively, after the Washington Stay Home Order was enacted [9]. Owing to the partial lockdown of São Paulo, Brazil, the CO concentration in urban areas decreased by 64.8% compared with the average of the past 5 years; the NO and NO_2_ concentrations along urban roads decreased by 77.3% and 54.3%, respectively; and the ozone concentration increased [10]. In Barcelona and Madrid, Spain, radical decreases in traffic owing to the lockdown in March 2020 resulted in a decrease in NO_2_ concentrations [11]. In Bari, Italy, NO_2_ concentrations decreased by 22.5% in March 2020, compared with that in March 2019 [12]. During the lockdown period in Almaty, Kazakhstan, concentrations of PM_2.5_, CO, and NO_2_ decreased by 21%, 49%, and 35%, respectively, compared with 2019. However, the concentration of benzene and toluene was 2–3 times higher than that in 2015–2019 [13]. Following 4 days of lockdown in 2020, India experienced a 40–50% improvement in PM_10_ and PM_2.5_ concentrations, compared with 2019 [14]. Lockdowns in some areas of China significantly improved the air quality, compared with the cities in which lockdown was not implemented [15]. Among them, in the Yangtze River Delta (YRD) region, as human activities decreased, SO_2_, NO_x_, PM_2.5_, and VOC concentrations decreased by 16–26%, and the ozone concentration increased [16]. A full lockdown in Shanghai resulted in reductions in PM_10_, PM_2.5_, CO, NO_2_, and SO_2_ of 34–48% in roadside stations and 18–50% in non-roadside stations [17]. In South Korea, the average concentrations of PM_2.5_, PM_10_, NO_2_, and CO in March 2020 decreased by 45.45%, 35.56%, 20.41%, and 17.33%, respectively, compared with those in March 2019 [18]; another study found that the average concentration of PM_2.5_ in February and March of 2020 decreased by 21%, compared with that in February and March of 2017–2019 owing to COVID-19 [19]. In Seoul, South Korea, 30-day average concentrations of PM_2.5_, NO_2_, and CO after social distancing (from 29 February to 29 March 2020) decreased by 10.4%, 16.4%, and 16.9%, respectively, compared with before the social distancing (from 30 January to 28 February 2020) [20]. In Seoul and Daegu, South Korea, the average concentrations of PM_2.5_ in March 2020 decreased by 36% and 30%, respectively, compared with those in March of 2017–2019 [21].

To the best of our knowledge, most of the previous studies estimated the effect of COVID-19 on air pollutant concentrations by comparing the concentrations before and after the outbreak of the COVID-19 pandemic. Only two previous studies further excluded climate- and policy-driven impacts from the total reduction in order to estimate COVID-19-driven reductions more accurately [9,19]. However, all of these studies were conducted without demonstrating the extent to which the reduction can actually be attributed to COVID-19. Therefore, the present study aims to examine the impact of COVID-19 on air quality by studying the variation in traffic volume. Traffic volume is one of the most known routes through which COVID-19 impacts air quality. This study identifies the relationship between traffic and air quality during 2016–2019. Subsequently, we study the relationship between the number of COVID-19 cases and traffic after 2020 in Seoul, South Korea.

## 2. Materials and Methods

### 2.1. Data Collection

The daily average concentrations of six major air pollutants (PM_10_, PM_2.5_, CO, NO_2_, O_3_, and SO_2_) in Seoul, South Korea, between January 2016 and June 2021 were obtained from the Korea Environment Corporation (https://www.airkorea.or.kr/web (accessed on 31 July 2021)). These values were calculated by averaging the concentrations measured every hour from each of the 25 urban monitoring stations located in Seoul.

The daily average weather data on the temperature, precipitation, and wind speed in Seoul between January 2016 and June 2021 were obtained from the Korea Meteorological Administration (https://data.kma.go.kr/cmmn/main.do (accessed on 31 July 2021)). They were calculated by averaging the data measured every hour from the single official station located in the center of Seoul.

Daily mobility data in Seoul between February 2020 and June 2021 were obtained from the Google Mobility database. These data show the percentage change in Seoul traffic compared with the median traffic between 2 January 2020 and 6 February 2020, in six categories: grocery and pharmacy, parks, transit stations, residential, workplaces, and retail and recreation [22]. The detailed information about these categories is available on the Google Mobility website (https://www.google.com/COVID19/mobility/ (accessed on 31 July 2021)).

The daily traffic volume (number of cars) on four arterial roads in Seoul between January 2016 and June 2021 was obtained from the Seoul Transport Operation and Information Service. These four arterial roads were the Dongbu Expressway, Gangbyeon Expressway, Gyeongbu Expressway, and Naebu Expressway, which run through Seoul, vertically and horizontally (see Figure S1 for a map of these arterial roads).

The daily number of new COVID-19 cases in South Korea from January 2020 to June 2021 was obtained from Our World in Data (https://ourworldindata.org/COVID-cases (accessed on 31 July 2021)). We chose the data to represent the severity of the COVID-19 situation, and because these data are widely available (not only for South Korea, but also for all countries across the world). We studied the COVID-19 cases for the whole of South Korea, not just those in Seoul, since the number of COVID-19 cases outside Seoul also affected mobility and traffic in Seoul. For example, during the first surge of COVID-19 cases in March of 2020 in Daegu, the traffic in Seoul was reduced even though Daegu is ~240 km away, and even before any social distancing measures were enacted.

Information on social distancing measures enacted in South Korea from January 2020 to June 2021 was obtained from the website managed by the Ministry of Health and Welfare in South Korea (http://ncov.mohw.go.kr/tcmBoardList.do?brdId=3&brdGubun= (accessed on 31 July 2021))

### 2.2. Regression Analysis

COVID-19 restrictions affected traffic volume and, in turn, affected the air quality. To quantitatively understand this chain of effects, we performed two regression analyses. We first extracted the relationship between the number of new COVID-19 cases and the percentage change in traffic from 2020 to 2021 in Seoul. In the present study, we chose the number of new COVID-19 cases rather than the social distancing policy as an independent variable. This was because the former is a quantitative variable, while the latter is a qualitative variable. The second analysis extracted the relationship between the percentage change in the traffic and air quality from 2016 to 2019 in Seoul. The data from after 2020 were not included in the second analysis in order to reveal the association before the COVID-19 pandemic. In these analyses, we removed outliers (data points located outside the average ± 3 standard deviations) from the air pollutant concentration and traffic data to ensure that they did not influence the regression results. We used R software version 4.0.4 and the “gam” function in the “mgcv” package to use a generalized additive model (GAM) [23]. GAM is commonly applied when using penalized splines, which help modeling nonlinearity without overfitting. [24].

In the first regression analysis, the association between COVID-19 and the percentage change in traffic was extracted using GAM with the assumption of a Gaussian distribution. The model used in this analysis is as follows:

(1) $$\begin{array}{ll} E\left[Traffic\right]= & \beta_{1}COVID19+s\left(Temperature,\;k=5\right)\\ & +s\left(Precipitation,\;k=5\right)+\beta_{2}DOW+s\left(Date,\;k=6\right)\\ & +\beta_{3}Holiday \end{array}$$

where $E\left[Traffic\right]$ is the expected daily traffic volume of the four expressways in Seoul; $\beta_{i}$ represents the regression coefficients; COVID19 represents the daily number of new COVID-19 cases in South Korea; $s\left(Temperature,\;k=5\right)$ and $s\left(Precipitation,\;k=5\right)$ are the smooth functions of the temperature and precipitation to control their effects on the traffic (we chose temperature and precipitation because they are the most basic meteorological factors); parameter k represents the number of basis functions used in the model (it was set to 5 to prevent overfitting); DOW is the day of the week to control the daily variation; $s\left(Date,\;k=6\right)$ is a smooth function of the date with 6 basis functions to control the seasonal variations (the number of basis functions were chosen to be equal to the number of seasons from January 2020 to June 2021); and Holiday is a categorical variable that indicates whether it is a non-holiday, a holiday on weekdays, or a holiday on weekends to control the extraordinary traffic patterns on holidays.

In the second regression analysis, we extracted the association between the percentage change in traffic and air pollutant concentrations using GAM with the assumption of a Gaussian distribution. The model used in this analysis is as follows:

(2) $$\begin{array}{ll} E\left[Conc.\right]= & \gamma_{1}Traffic+s\left(Temperature,\;k=5\right)+s\left(Precipitation,\;k=5\right)\\ & +s\left(WindSpeed,\;k=5\right)+\gamma_{2}DOW+s\left(DOY,\;k=4\right)\\ & +s\left(Date,\;k=2\right) \end{array}$$

where $E\left[Concentration\right]$ is the expected daily concentration of pollutants in Seoul; $\gamma_{i}$ represents the regression coefficients; Traffic is the daily traffic volume at the 4 expressways in Seoul; $s\left(WindSpeed,\;k=5\right)$ is a smooth function of the wind speed functions to control the effect of wind on air pollutant concentrations; to temperature and precipitation, we added wind speed, since wind can affect pollutant concentrations via mixing; $s\left(DOY,\;k=4\right)$ is a smooth function of the day of year (DOY, from day 1 to day 365) with 4 basis functions to control the seasonal variation within a year (the number of basis functions was set to 4, which is identical to the number of seasons in a year—this method of modeling seasonal variability has been widely used in previous studies [25,26,27,28]); and $s\left(Date,\;k=2\right)$ is a smooth function of the date to control long-term variations, which occur over a few years. By setting *k* = 2, $s\left(Date,\;k=2\right)$ can only model the long-term variations.

### 2.3. Calculation of COVID-19-Attributable Air Quality Change

The relationship between COVID-19 cases and air pollutant concentrations through traffic reduction can be calculated based on the coefficients obtained from the two regression analyses as follows:

(3) $$\frac{Concentration\;changes}{\mathrm{COVID}-19\;cases}=\frac{traffic\;changes}{\mathrm{COVID}-19\;cases}\cdot \frac{concentration\;changes}{traffic\;changes}$$

Then, the COVID-19-attributable change (CAC) in air quality can be calculated by

(4) $$CAC=\frac{Concentration\;changes}{\mathrm{COVID}-19\;cases}\cdot Mean\;\mathrm{COVID}-19\;cases$$

In addition, the COVID-19-attributable change fraction (CACF) in air quality can be calculated as:

(5) $$CACF=\frac{CAC}{Mean\;air\;pollutant\;concentration}$$

## 3. Results

### 3.1. Association of Traffic with COVID-19

**Figure 1:**
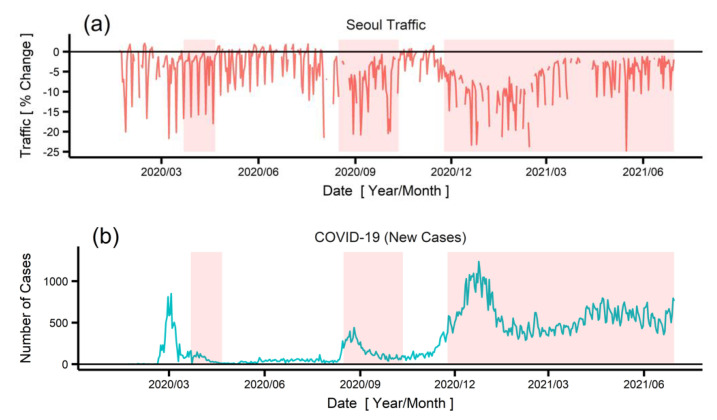
(**a**) Daily traffic changes in Seoul, South Korea (reference value is median traffic volume between 2016 and 2021), and (**b**) new COVID-19 cases in South Korea, between January 2020 and June 2021. Areas shaded in red show the periods when enhanced social distancing measures were applied.

**Figure 2:**
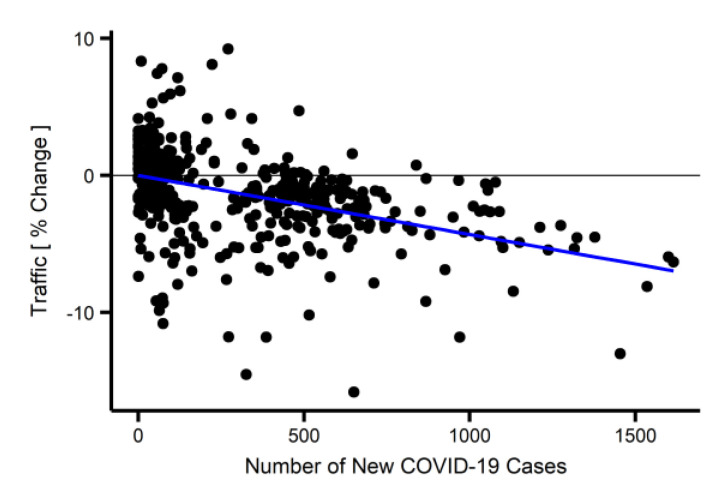
Relationship between the number of new COVID-19 cases and the percentage change in traffic from January 2020 to June 2021. Black dots show the daily relationship, and the blue solid line represents the linear regression.

Figure 1 shows the percentage change in the traffic measured at four expressways in Seoul and the number of new COVID-19 cases in South Korea since 2020. The baseline of the traffic data was the median traffic volume between 2016 and 2021 (see Figure S2 for the traffic volume of the four expressways separately). The discontinuous points in the traffic data were either missing from the original data or outliers that were excluded from the analysis. The periodic spikes observed in the traffic data were caused by weekends and holidays. Note that a decrease in Seoul traffic corresponds to a surge in COVID-19 cases. There were approximately four surges, those in around March 2020, August 2020, December 2020, and June 2021. During these surge periods, the Seoul traffic volume decreased; the scale of the decrease was especially high in the second and third surges. This was partly because of the increased level of social distancing policies applied during the second and third surge periods, and the difference in people’s perceptions about the severity of the COVID-19 pandemic. Figure 2 shows the relationship between the number of COVID-19 cases and the percentage change in traffic based on the GAM. The blue curve, which shows the linear regression result, clearly demonstrates that the traffic volume decreased with the increase in COVID-19 cases (coefficient of −0.00431%/case). This means that the traffic volume reduced by 0.431% each increase of 100 new COVID-19 cases.

In addition to the association analysis between the traffic and COVID-19 cases, based on Google Mobility data, we analyzed the categories of peoples’ movements that were most affected by COVID-19. Figure S3 shows the Google Mobility data and new daily cases of COVID-19. Several spikes observed in the mobility data coincided with holidays. Among the six categories of mobility, retail and recreation, transit stations, and workplaces had strong negative correlations with the COVID-19 cases, while mobility in residential areas showed a strong possible correlation with COVID-19 cases. This was partly because social distancing policies were strengthened with the surge, the number of people working from home increased, and people became more reluctant to use public transportation and retail stores. Unlike other categories, the mobility to grocery and pharmacy were not significantly impacted by the surge of COVID-19 cases since these are necessities; the relationship between mobility to parks and COVID-19 cases remains unclear owing to large seasonal variations.

### 3.2. Association of Air Pollutant Concentrations with Traffic

**Figure 3:**
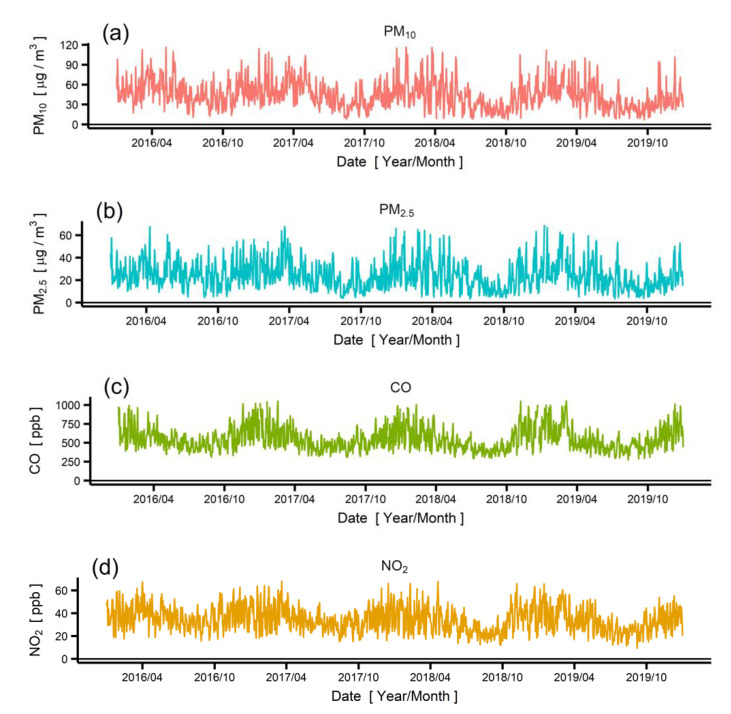

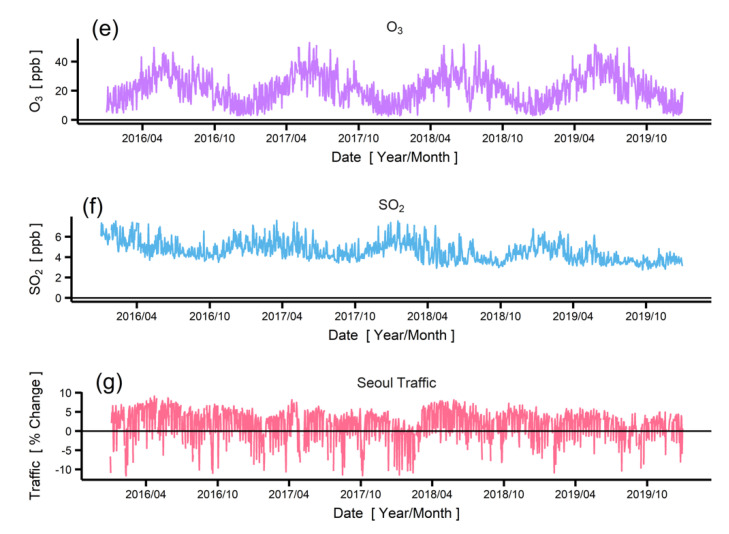
(**a**–**f**) Time-series plots of pollutant concentration data in Seoul: (**a**) PM_10_, (**b**) PM_2.5_, (**c**) CO, (**d**) NO_2_, (**e**) O_3_, and (**f**) SO_2_. (**g**) Percentage change in Seoul traffic between 2016 and 2019.

Figure 3 shows the air pollutant concentrations and the percentage change in the expressway traffic volume in Seoul from 2016 to 2019 (Figure S2 shows the separate traffic volumes of each expressway). As observed from the time-series plot, the air pollutant concentrations exhibited significant daily and seasonal variations. These variations are mostly due to daily and seasonal variations in weather conditions, which determine the rate of spatial mixing and pollutant formation. To extract the relationship between the traffic and pollutant concentrations, we used GAM with various confounding factors, as shown in Equation (2).

**Figure 4:**
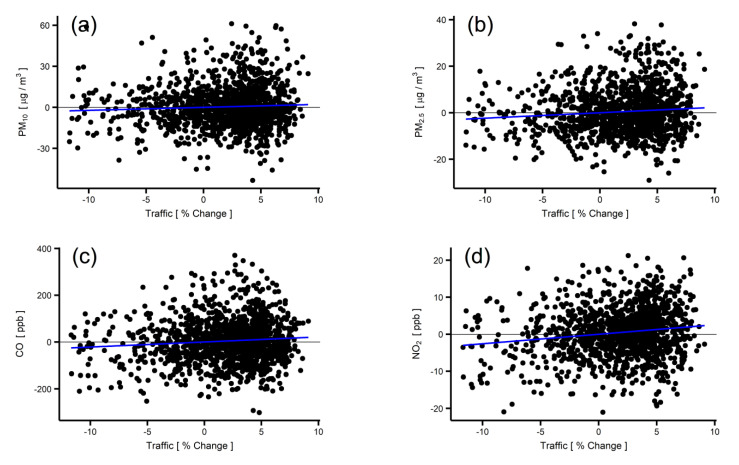

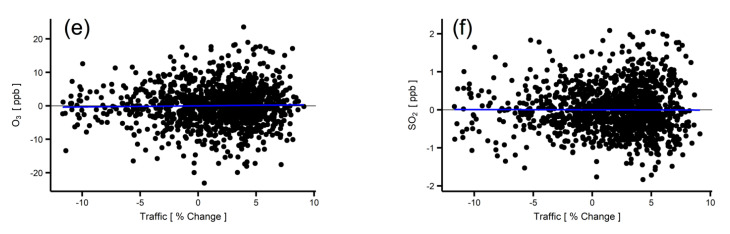
(**a**–**f**) Relationship between pollutant concentrations and the percentage change of traffic: (**a**) PM_10_, (**b**) PM_2.5_, (**c**) CO, (**d**) NO_2_, (**e**) O_3_, and (**f**) SO_2_. Black dots show the daily relationship between these parameters from 2016 to 2019; the blue solid lines represent the linear regressions.

Figure 4 shows the relationship between traffic change and air pollutant concentrations based on the GAM; the blue lines show the linear regression results. Table 1 summarizes the coefficients of the regressions, along with the mean pollutant concentrations. The ratio in Table 1 shows the percentage of air pollutant change with a 1% change in traffic. Our results show that a 1% change in traffic resulted in the largest variation, in PM_2.5_ (0.94%), followed by a variation in NO_2_ (0.74%). Unlike other pollutants, the concentration of SO_2_ was not significantly affected by traffic (−0.01%). Table S1 shows the fitness test of the models and the significance test of the traffic factor. The models for CO, NO_2_, and O_3_ showed a relatively better fit (R^2^ of 0.62–0.70), while the models for PM_10_ and PM_2.5_ showed a relatively poorer fit (R^2^ of 0.37–0.44). The *p*-values for PM_2.5_, CO, and NO_2_ were less than 0.05, rejecting the null hypothesis, while the *p*-values for O_3_ and SO_2_ indicated that the relationships between traffic volume and concentrations of O_3_ and SO_2_ were week (if any).

### 3.3. COVID-19-Attributable Air Quality Changes via Traffic Reduction

The increase in COVID-19 cases reduced the traffic volume and, in turn, improved the air quality. Based on the two regression results, we calculated the extent to which the changes in air quality may have been attributable to traffic reduction caused by the COVID-19 pandemic. Table 2 shows the CAC and CACF changes in the air quality due to traffic reduction in Seoul from 2020 to 2021 (see Section 2.3). The COVID-19 pandemic affected the concentrations of PM_10_, PM_2.5_, CO, NO_2_, and O_3_ by −0.67%, −1.33%, −0.54%, −1.20%, and −0.16%, respectively, while it had little impact on the SO_2_ concentration.

### 3.4. Projection of Air Pollutant Concentrations in 2020–2021

**Figure 5:**
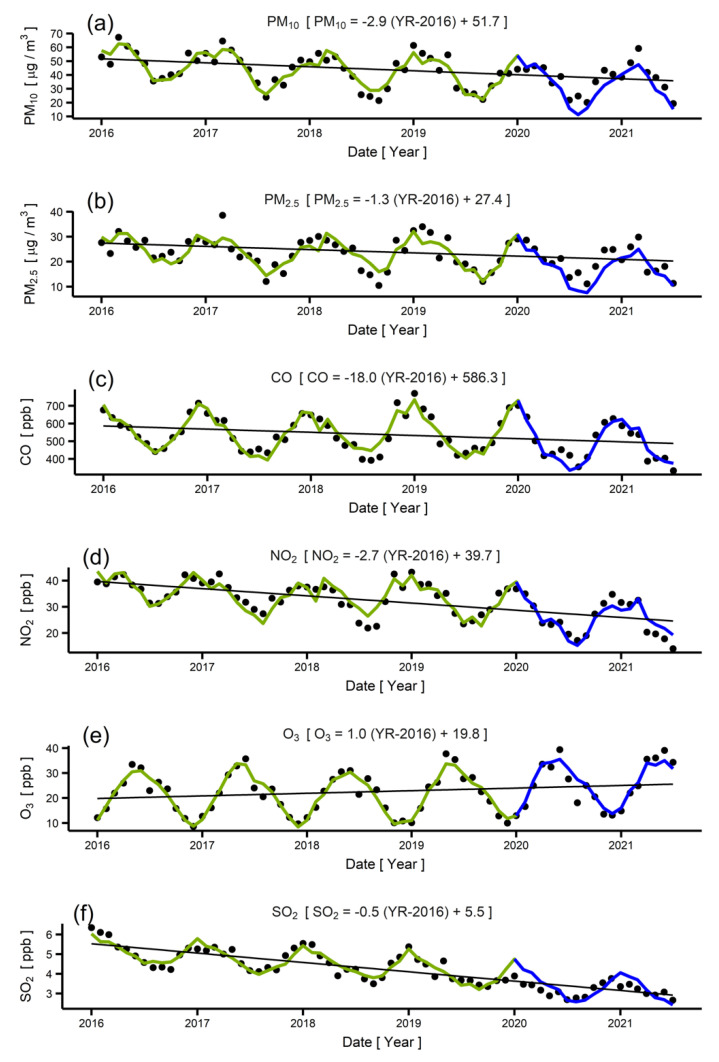
(**a**–**f**) Measured concentrations (black points) and predicted concentrations (blue lines) of pollutants in 2020–2021 based on the extracted models (green lines) of 2016–2019: (**a**) PM_10_, (**b**) PM_2.5_, (**c**) CO, (**d**) NO_2_, (**e**) O_3_, and (**f**) SO_2_. Equations in brackets describe the linear long-term trends shown in black lines.

Figure 5 shows the measured monthly concentrations (black points) of various air pollutants in Seoul in 2016–2021, along with the modeled concentrations (green lines) in 2016–2019 and predicted concentrations (blue lines) from 2020 to 2021. Since the model used in the prediction was extracted based on the data from 2016 to 2019 (before the COVID-19 pandemic), only the traffic-associated impact of COVID-19 was included in the prediction. Table 3 shows numerical comparisons between the measured, modeled, and predicted yearly pollutant concentrations. We observed deviations between the measured and predicted concentrations in 2020–2021. Such deviations may partly stem from the simplicity of the model in Equation (2) and the variability of the yearly reduction trend. However, despite such an uncertainty, there is no point of inflection in long-term concentration trends near initial outbreak of COVID-19 (December 2019). Rather, it appears that most of the concentration reduction in 2020–2021, compared with that in 2016–2019, was not due to COVID-19, but instead reflects a long-term trend of a reduction in air pollutants.

## 4. Discussion

In this study, the traffic-associated impacts of COVID-19 on the air pollutant concentrations of PM_10_, PM_2.5_, CO, NO_2_, and O_3_ were −0.67%, −1.33%, −0.54%, −1.20%, and −0.16%, respectively, in Seoul during 2020–2021. These reductions are much smaller than those demonstrated by Ju et al. [15], Kang et al. (2021) [16], Han et al. [17], and Seo et al. [18]. These differences reflect two main factors. First, the present study focused only on traffic-related impacts that are 100% attributable to COVID-19, while previous studies compared concentration differences before and after the outbreak of COVID-19 pandemic. The approach taken by the present study may underestimate the total impact of COVID-19 by not considering other possible routes that may relate COVID-19 to air pollutant concentrations, while the approach taken by the previous studies may overestimate the total impact of COVID-19 by not successfully excluding all other reduction causes. Second, the present study estimated the impact of COVID-19 on 18-month-average (January 2020 to June 2021) concentrations of air pollutants, while previous studies focused on the impacts on concentrations over 1 or 2 months after a COVID-19 outbreak or social distancing.

There are a few limitations to this study. First, we used the non-linear regression model in Equation (2) to rule out the confounding effects of climate when extracting the relationship between traffic and air pollutant concentrations. Although this is a widely used method to exclude the confounding effects of climate, there may be a possibility to improve the accuracy of the relationship by using models based on atmospheric science and physics, such as Community Multiscale Air Quality (CMAQ). Second, the deviance of pollutant concentrations explained by traffic volume is small (see Figure 4). This implies that there may be other confounding factors (e.g., industrial emissions, long-range transport from other countries, and other meteorological parameters) in the relationship between traffic and pollutant concentrations (Figure 4). A successful consideration of such confounding factors could improve the accuracy of the analysis. Third, the relationship between traffic and pollutant concentrations is not reliable outside the range of historical data points (±10% of traffic change). Lastly, we assumed that the traffic along four expressways, climate data measured by the single official site in Seoul, and the air quality data measured by 25 official sites spread in Seoul can represent the total traffic, climate, and air-quality conditions in Seoul. Any inaccuracy originating from this assumption may limit the accuracy of the analysis.

We will address these limitations in future work. In addition, we also plan to focus on other possible routes where COVID-19 impacted on air quality, including the reduction in anthropogenic emissions due to economic slowdown driven by COVID-19-related lockdowns or socio-economic changes, such as unemployment.

## 5. Conclusions

This study analyzed the change in the concentrations of air pollutants as a function of the number of confirmed COVID-19 cases. COVID-19-attributable air quality improvements through traffic reduction were found to be 0.67%, 1.33%, 0.54%, 1.20%, and 0.16% for PM_10_, PM_2.5_, CO, NO_2_, and O_3_, respectively, in Seoul during 2020–2021. This study is the first to demonstrate the traffic-associated impacts of COVID-19 on air quality, and the methodology used in the study could be further applied to quantify the real contribution of COVID-19 on air quality changes.

## Figures and Tables

**Table 1:** Coefficients of linear regressions between the traffic and pollutant concentrations based on the generalized additive model (GAM), along with mean pollutant concentrations in Seoul from 2016 to 2019. The ratio shows the percentage change in the pollutant concentrations with 1% change in traffic.

Pollutant	Coefficient	Mean Concentration	Ratio
PM_10_	0.219 µg/m^3^/%	45.5 µg/m^3^	0.48%
PM_2.5_	0.233 µg/m^3^/%	24.9 µg/m^3^	0.94%
CO	2.161 ppb/%	553.8 ppb	0.39%
NO_2_	0.257 ppb/%	34.6 ppb	0.74%
O_3_	0.033 ppb/%	21.5 ppb	0.16%
SO_2_	−0.0007 ppb/%	4.6 ppb	−0.01%

**Table 2:** COVID-19-attributable change (CAC) and change fraction (CACF) in air quality due to traffic reduction in Seoul from 2020 to 2021. Concentration changes per COVID-19 case and mean concentration during 2020–2021 are also shown. In the CAC calculation, the daily mean COVID-19 cases of 288.3 people during 2020–2021 were used.

Pollutant	Concentration Change Per COVID-19 Case	Mean Concentration	COVID-19-Attributable Air Quality	
			CAC	CACF
PM_10_	−0.00094 μg/m^3^/case	40.4 μg/m^3^	−0.27 μg/m^3^	−0.67%
PM_2.5_	−0.00100 μg/m^3^/case	21.8 μg/m^3^	−0.29 μg/m^3^	−1.33%
CO	−0.00931 ppb/case	501.7 ppb	−2.68 ppb	−0.54%
NO_2_	−0.00111 ppb/case	26.6 ppb	−0.32 ppb	−1.20%
O_3_	−0.00014 ppb/case	25.3 ppb	−0.04 ppb	−0.16%
SO_2_	0.000003 ppb/case	3.2 ppb	0.00 ppb	0.02%

**Table 3:** Comparison between the measured (2016–2021), modeled (2016–2019), and predicted (2020–2021) pollutant concentrations.

Type	Pollutant (Unit)	2016	2017	2018	2019	2020	2021
Measured	PM_10_ (µg/m^3^)	49.4	45.5	40.5	41.0	37.0	41.8
	PM_2.5_ (µg/m^3^)	25.9	23.2	22.9	23.4	21.1	20.8
	CO (ppb)	569.8	537.8	534.1	551.6	510.9	474.6
	NO_2_ (ppb)	37.7	35.0	32.6	32.9	27.2	25.2
	O_3_ (ppb)	21.0	21.3	20.8	22.6	23.1	28.8
	SO_2_ (ppb)	5.2	4.8	4.4	4.0	3.3	3.2
Modeled (2016–2019) and predicted (2020–2021)	PM_10_ (µg/m^3^)	49.2	44.3	42.6	39.9	33.5	36.5
	PM_2.5_ (µg/m^3^)	25.7	22.9	23.9	22.4	17.8	19.1
	CO (ppb)	569.9	526.3	553.9	539.4	493.4	496.2
	NO_2_ (ppb)	38.0	33.3	34.6	32.1	26.3	26.6
	O_3_ (ppb)	20.9	21.6	20.4	22.7	24.5	27.8
	SO_2_ (ppb)	5.1	4.8	4.5	4.0	3.4	3.3
Deviation	PM_10_ (µg/m^3^)	−0.4%	−2.7%	5.2%	−2.7%	−9.5%	−12.7%
	PM_2.5_ (µg/m^3^)	−0.8%	−1.3%	4.6%	−4.4%	−16.0%	−7.8%
	CO (ppb)	0.0%	−2.1%	3.7%	−2.2%	−3.4%	4.5%
	NO_2_ (ppb)	0.9%	−5.0%	6.3%	−2.6%	−3.2%	5.7%
	O_3_ (ppb)	−0.6%	1.7%	−1.9%	0.3%	6.3%	−3.3%
	SO_2_ (ppb)	−0.8%	−1.0%	0.8%	0.0%	5.7%	5.5%

## Data Availability

The datasets used and/or analyzed during the current study are available from the corresponding author on reasonable request.

## Supplementary Materials

The following are available online at https://www.mdpi.com/article/10.3390/ijerph19031718/s1, Figure S1: Map of Seoul with four expressways; Figure S2: Daily traffic volume at four expressways in Seoul from 2016 to 2021; Figure S3: (a)–(f) Google Mobility data in Seoul: (a) Grocery and Pharmacy, (b) Parks, (c) Residential, (d) Retail, (e) Transit, (f) Workplaces. (g) the number of new COVID-19 cases in South Korea between February 2020 and June 2021; Table S1: Fitness test (R2) of the models and significance test of the traffic factor.
