# Supplementary material for: Impacts of COVID-19 on Air Quality through Traffic Reduction
Source: Int J Environ Res Public Health. 2022 Feb 2;19(3):1718. doi: 10.3390/ijerph19031718 (PMC8834776; doi:10.3390/ijerph19031718)
Supplement: Supplementary file 1 [file ijerph-19-01718-s001.zip › ijerph-1531866-supplementary.pdf]

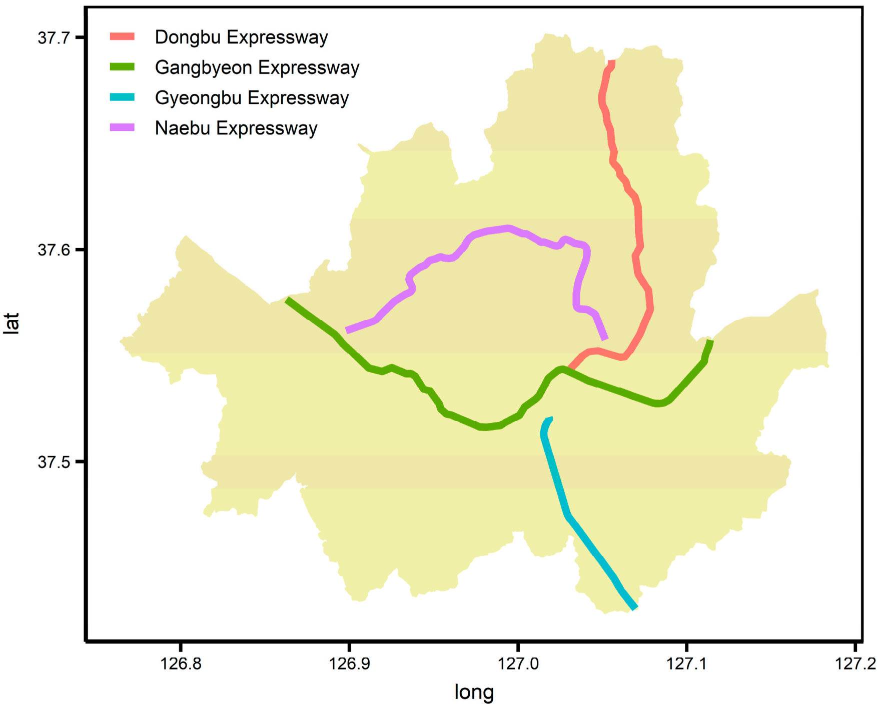

Figure S1. Map of Seoul with four expressways.

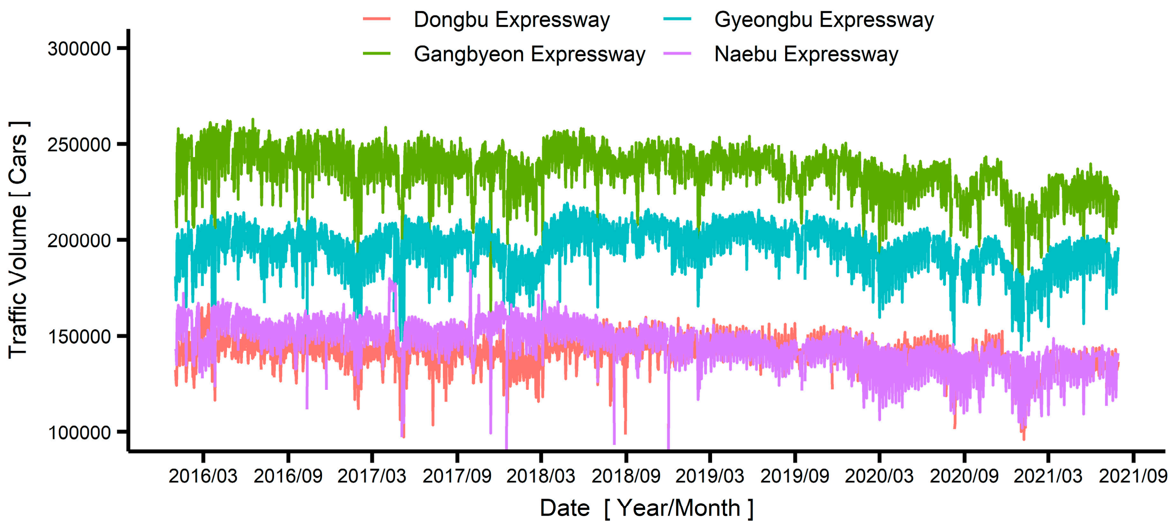

Figure S2. Daily traffic volume at four expressways in Seoul from 2016 to 2021.

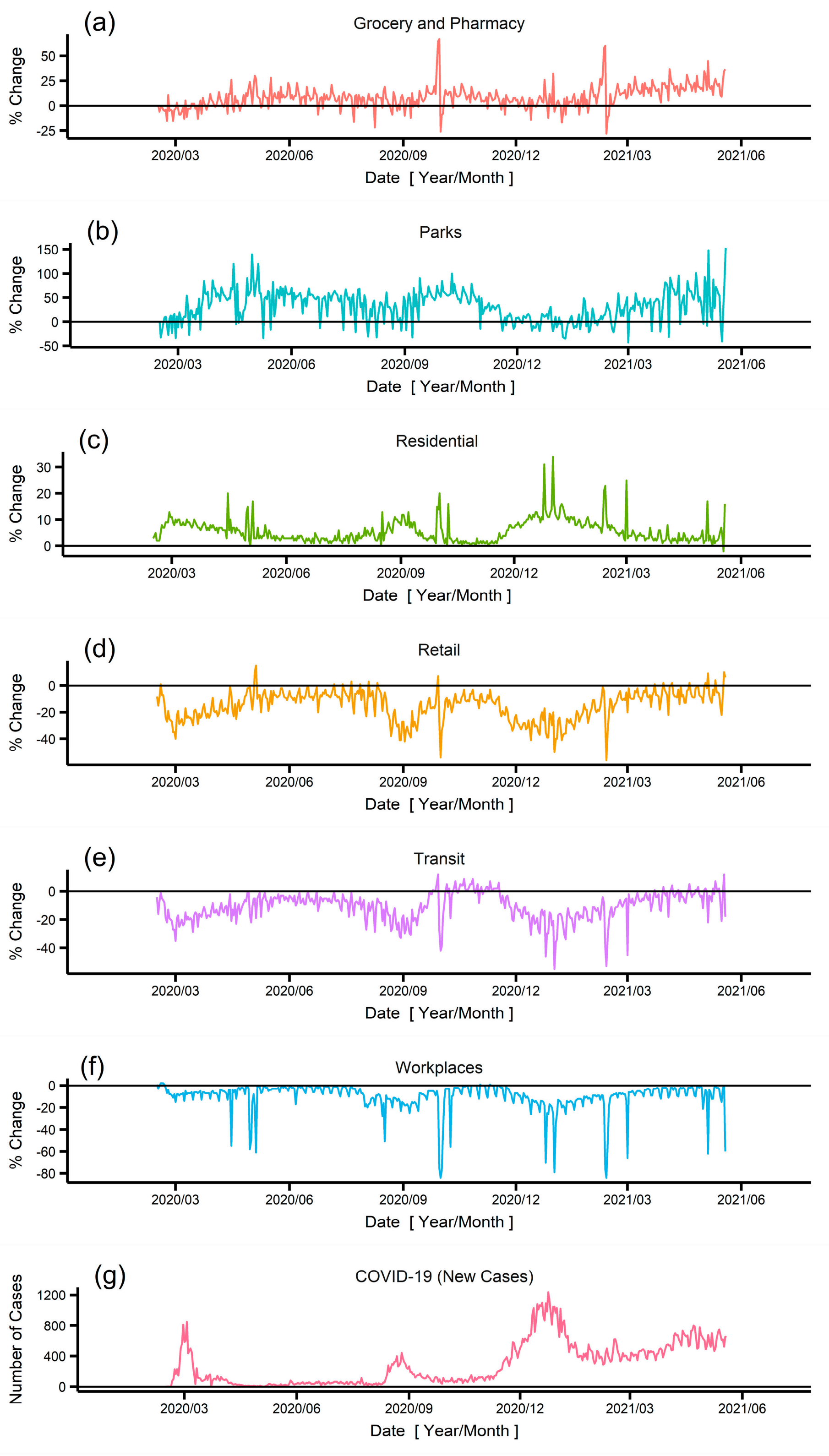

Figure S3. (**a**)–(**f**) Google Mobility data in Seoul: (**a**) Grocery and Pharmacy, (**b**) Parks, (**c**) Residential, (**d**) Retail, (**e**) Transit, (**f**) Workplaces. (**g**) the number of new COVID-19 cases in South Korea between February 2020 and June 2021.

Table S1. Fitness test (R^2) of the models and significance test of the traffic factor.

	R^2 of the Model	Traffic		
		Coefficient	Std. Error	P-value
PM_{10}	0.44	0.219 μg/m^3/%	0.157 μg/m^3/%	0.162
$PM_{2.5}$	0.37	0.233 μg/m^3/%	0.099 μg/m^3/%	0.018
CO	0.62	2.161 ppb/%	0.955 ppb/%	0.023
NO_2	0.64	0.257 ppb/%	0.066 ppb/%	0.000
O_3	0.70	0.033 ppb/%	0.057 ppb/%	0.560
SO_2	0.59	-0.0007 ppb/%	0.006 ppb/%	0.913
